# A Reduced Three Dimensional Model for SAW Sensors Using Finite Element Analysis

**DOI:** 10.3390/s91209945

**Published:** 2009-12-08

**Authors:** Mohamed M. El Gowini, Walied A. Moussa

**Affiliations:** Department of Mechanical Engineering, University of Alberta, Edmonton, AB, T6G 2G8, Canada; E-Mail: elgowini@ualberta.ca

**Keywords:** Finite Element Analysis, Aluminum Nitride, Surface Acoustic Waves, MEMS, SAW sensors

## Abstract

A major problem that often arises in modeling Micro Electro Mechanical Systems (MEMS) such as Surface Acoustic Wave (SAW) sensors using Finite Element Analysis (FEA) is the extensive computational capacity required. In this study a new approach is adopted to significantly reduce the computational capacity needed for analyzing the response of a SAW sensor using the finite element (FE) method. The approach is based on the plane wave solution where the properties of the wave vary in two dimensions and are uniform along the thickness of the device. The plane wave solution therefore allows the thickness of the SAW device model to be minimized; the model is referred to as a Reduced 3D Model (R3D). Various configurations of this novel R3D model are developed and compared with theoretical and experimental frequency data and the results show very good agreement. In addition, two-dimensional (2D) models with similar configurations to the R3D are developed for comparison since the 2D approach is widely adopted in the literature as a computationally inexpensive approach to model SAW sensors using the FE method. Results illustrate that the R3D model is capable of capturing the SAW response more accurately than the 2D model; this is demonstrated by comparison of centre frequency and insertion loss values. These results are very encouraging and indicate that the R3D model is capable of capturing the MEMS-based SAW sensor response without being computationally expensive.

## 1. Introduction

Surface Acoustic Wave (SAW) devices are considered to be one of the early examples of Micro-Electromechanical systems (MEMS) [1] due to the coupling needed between electrical and mechanical properties during wave propagation on the surface of these devices. These devices' high reliability and relative simplicity in fabrication and integration motivated MEMS researchers to utilize it in a broad range of applications such as TVs, VCRs, radar systems, wireless headsets, alarm systems and mobile phones. In addition, the propagation of the wave along the surface allows it to be sensitive to changes in the external environment; therefore SAW sensors have been developed for numerous applications such as gas detection [2], fluid viscosity changes [3], and pressure changes [4], determination of stiffness constants [5] and detection of the onset of ice formation on aerospace structures [6].

The design process for SAW devices is highly iterative due to the various parameters that could be manipulated to utilize its sensitivity; such as electrode dimensions, size, shape and configuration, piezoelectric substrate material, waveguide material and dimensions, operating frequency and mode of wave propagation. In addition, there is a wide range of complex electromechanical interactions that take place in a typical SAW device. To optimize the design phase various numerical and analytical techniques have been developed and some are used concurrently.

The Delta function model is one of the earliest and basic modeling techniques of SAW devices. This model provides a basic understanding of the response of SAW devices. It only provides relative insertion loss since it does not take into consideration impedance level and second order effects [7]. However, this model provides very good information on bandwidth, rejection levels and side lobes. When a voltage signal is applied to the inter-digital (IDT) electrodes, there is an instantaneous charge accumulation. Due to the alternating polarity of adjacent electrodes the charges accumulate towards the edges of an electrode. This model represents the charge distribution on the surface of the electrodes as discrete delta functions. The magnitude of the delta functions is proportional to the amplitude of the applied voltage signal. The total response of an IDT due to an applied voltage signal is obtained by summing the delta functions on the electrodes.

**Figure 1.**
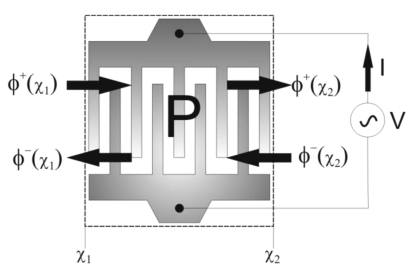
Representation of an IDT structure as a three port network.

The Coupling of Modes (COM) approach is another technique used to model SAW devices. This technique branches out of the general wave propagation field in periodic structures, which covers a wide range of wave phenomena such as electromagnetic waves in periodic gratings, optical and ultrasonic waves in multi-layered media, phonon propagation and X-ray scattering in crystals, quantum theory of electron states in metals, semiconductors and dielectrics [8]. The coupling of modes approximation indicates that in periodic structures only the incident wave and the reflected wave with strong coupling are considered [8]. The two waves are counter-propagating and a linear coupling is assumed between the amplitudes, voltage and current. The spatial variation of the amplitude of the two modes and the current generated in the conducting electrodes are described through a set of first order linear differential equations. The COM equations are often represented, for convenience by the P-matrix method developed by Tobolka [9]. The P-matrix represents an IDT structure as a three port network with two acoustical ports and a third electrical port. The coefficients of the P-Matrix are determined from the COM parameters; velocity, reflectivity, transduction coefficient, attenuation and capacitance. Figure 1 illustrates an IDT structure as a three port network. The boundary conditions are the applied voltage *V* and the incident waves with amplitudes *φ*^+^(*x*_1_) and *φ*^−^(*x*_2_), respectively. The response of the device is represented by the current (*I*) generated at the electrodes and the reflected waves with amplitudes *φ*^−^(*x*_1_) and *φ*^+^(*x*_2_).

The P-matrix representation of an IDT structure relating the boundary conditions to device response is given by:

(1) $$\left[\begin{array}{c} \phi^{-}(x_{1})\\ \phi^{+}(x_{2})\\ I \end{array}\right]=\left(\begin{array}{ccc} P_{11} & P_{12} & P_{13}\\ P_{21} & P_{22} & P_{23}\\ P_{31} & P_{32} & P_{33} \end{array}\right)\left[\begin{array}{c} \phi^{+}(x_{1})\\ \phi^{-}(x_{2})\\ V \end{array}\right]$$

The upper left sub-matrix describes the scattering of the incident waves. The coefficients *P*_11_ = *P*_22_ are reflection coefficients and *P*_12_ = *P*_21_ are transmission coefficients. The remaining elements of the P-matrix represent the electrical properties of the device; *P*_13_ and *P*_23_ describe the electro-acoustic transfer function of the IDT. The components *P*_31_ and *P*_32_ determine the current generated in the IDT by the arriving waves. The *P*_33_ term is the admittance term, which relates the generated current to the applied voltage. One of the main advantages of the P-matrix method is its simplicity in modeling devices with different sub-structures. A separate P-matrix can be developed for each sub-structure of the SAW device and all can be cascaded into one P-matrix that represents the whole device.

The Equivalent Circuit model is another modeling technique, whose representation is close to the P-matrix. In this modeling technique, the equivalent circuit of the SAW device is developed and the IDT structure is modeled as a three port network [7]. Two ports are the electrical equivalent of the two acoustic ports in the P-matrix and the third port is an actual electrical port at which the input and output signals are applied and detected. The boundary conditions in this case are the applied voltages, while the response is considered to be current generation at the three ports. The boundary conditions are related to the response through the admittance matrix. The electrical parameters of the equivalent circuit are determined from wave and device properties such as wave velocity, substrate electromechanical coupling coefficient, centre frequency and number of electrode pairs. As in the P-matrix approach, an IDT with different sub-structures can be easily modeled by cascading the various admittance matrices for the sub-structures and then obtaining the overall transfer function of the SAW device from the ratio of output to input voltage.

The FE method can be used concurrently with the COM and equivalent circuit models. The COM parameters pertaining to the device configuration need to be determined to be used as input for these models. Test structures could be fabricated and analyzed in order to extract the necessary parameters. However, the experimental approach is both expensive and time consuming since the necessary parameters have to be extracted for each device configuration. On the other hand, the FE method provides an alternative approach for determining the device parameters in a time and cost efficient manner. The ability to model SAW devices is based on the well established theory of applying the FE method to piezoelectric vibration. The finite element formulation of piezoelectric media is provided at a later section in this article, however, a comprehensive review is also available in [10,11]. This numerical technique provides a greater flexibility in modeling SAW devices because it can handle the wave equations in two and three dimensions. This allows capturing the full device response and enhances the ability to model complex geometries and test different designs for optimum performance.

Various researchers have successfully modeled SAW sensors using the FE method to investigate different aspects of these devices such as sensor response to mass loading [12-14], various device configurations [15,16], power consumption evaluation [1] and mass sensitivity evaluation [17]. A common problem in modeling SAW devices is the increased computational capacity, which often arises due to the mandatory requirement of having a sufficient number of elements along the wavelength in the propagation path. This requirement ensures that the wave is fully captured and hence the sensor response is accurate. The operating frequency and wave velocity in SAW devices are relatively high, where the wavelengths are usually in the micrometer range, therefore the size of the elements have to be significantly reduced with respect to the wavelength to accurately capture the response. This leads to an increase in the overall number of elements in the FE model and hence increases the computational capacity. Various researchers have reported constraints due to the increased computational requirements of the FE models and made various attempts to reduce it [15,17-20]. Some of these attempts include reducing sensor dimensions, developing a two dimensional model and manipulating element sizes to reduce overall element count.

In almost all of the FE models referred to above, bulk piezoelectric substrates are adopted, such as lithium niobate (LiNbO_3_), quartz, langasite (LGS) and lithium tantalate (LiTaO_3_). The most common orientation of these substrates are Y-Z LiNbO_3_, ST-X Quartz, Z-X LiNbO_3_ and 36 Y-X LiTaO_3_ with the corresponding SAW velocities of 3,488 m/s [15], 3,159 m/s [21], 3,797 m/s [22] and 4,220 m/s [23], respectively. The limitation due to model size poses a greater obstacle to modeling new trends in SAW devices. Current development is heading towards producing a fully integrated system on one chip referred to as a Monolithic Chip [24]. The chip allows the integration of all system components in a single platform, reduction in size, low fabrication cost, mass production and low power consumption. Bulk piezoelectric materials are incompatible with planar integrated circuit technology; therefore, “layered” SAW devices are being developed. A layered SAW device consists of a silicon substrate covered by thin piezoelectric film. This configuration requires materials that can be deposited with high piezoelectric properties that closely match the corresponding single crystal properties. The two most widely used materials are Aluminum Nitride (AlN) and Zinc Oxide (ZnO) [25] since both materials possess exceptionally high piezoelectric properties. In addition, both can be deposited in a well oriented structure on a variety of substrates such as silicon, sapphire, diamond, graphite and glass. However, AlN is more widely adopted due to its higher resistivity and higher SAW velocity; 5,067 m/s [26]. This allows achieving much higher frequency levels than that attainable with bulk piezoelectric materials hence leading to even smaller wavelengths.

In this study, the FE method is used to develop an idealized model of a layered SAW sensor, with AlN used as the piezoelectric film on a silicon substrate. The idealized model is a “Reduced” 3D (R3D) model. The model assumes a plane wave solution, therefore, the wave properties vary in two dimensions only and is uniform in the third dimension; the thickness direction. This idealization allows minimizing the thickness of the sensor, which reduces the overall required number of elements. The results of the R3D model are compared to experimental and theoretical frequency data. In addition, the frequency response and insertion loss values of the R3D model are compared to those of 2D models with similar configuration.

## 2. Operating Principle of SAW Devices

**Figure 2.**
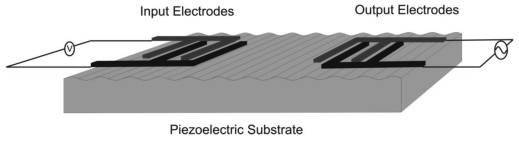
Layout of the SAW delay line structure.

The SAW device configuration adopted in this study is the delay line structure, where two sets of electrodes are patterned on the surface of the piezoelectric substrate. The electrodes are arranged in an inter-digital pattern as illustrated in Figure 2.

A voltage signal is applied at the input electrodes, which by the *converse* piezoelectric effect is converted to mechanical perturbations on the surface. The acoustic wave propagates in the area between the two sets of electrodes. As the wave reaches the output set of electrodes the mechanical wave is converted into an electrical signal by the *direct* piezoelectric effect.

### 2.1. Plane Wave Solution

Propagation of Surface Acoustic Waves in piezoelectric crystals is governed by the mechanical equation of motion and the electromagnetic field equations. The equations are coupled by the piezoelectric constitutive equations given by:

(2) $$\begin{array}{c} T_{\text{ij}}=c_{\text{ijkl}}^{E}S_{\text{kl}}-e_{\text{ijk}}^{T}E_{k}\\ D_{i}={ɛ}_{\text{ij}}^{S}E_{j}+e_{\text{ikl}}S_{\text{kl}} \end{array}$$

where *D* is the electric displacement field and has units (C/m^2^); *ε_ij_* are the dielectric permittivity constants and have units (F/m); *E_j_* is the electric field component and has units (V/m) and the constants *e_ijk_* and *e_ikl_* are the piezoelectric stress constants and have units (C/m^2^). The piezoelectric stress matrices in both equations are transposes of each other, hence the superscript (*T*). The piezoelectric matrix couples the electric and mechanical fields. The superscripts on the elastic stiffness constants and the dielectric permittivity constants imply that these are the properties at constant electric field and strain, respectively.

When solving for acoustic waves the magnetic field is assumed to be static and hence the electric field is assumed to be the gradient of the scalar potential:

(3) $$E_{i}=\frac{\partial \phi }{\partial r_{i}}$$

where *φ* is the electric potential. This is called the quasi static approximation and has negligible effect on the solution [16]. The equation of motion for a vibrating particle in the absence of body forces is:

(4) $$\sum_{j=1}^{3}\frac{\partial }{\partial r_{j}}T_{\text{ij}}=\rho \frac{\partial^{2}u_{i}}{\partial t^{2}}$$

where *ρ* is the particle density and *u_i_* is the displacement component in the *i^th^* direction. Substituting Equation (3) and Equation (4) in the first piezoelectric constitutive equation Equation (2) yields the first second order wave equation:

(5) $$c_{\text{ijkl}}^{E}\frac{\partial^{2}u_{k}}{\partial r_{j}\partial r_{l}}+e_{\text{ijk}}\frac{\partial^{2}\phi }{\partial r_{k}\partial r_{j}}=\rho \frac{\partial^{2}u_{i}}{\partial t^{2}}$$

Piezoelectric materials are insulators; therefore the absence of electric charge within the material can be expressed by:

(6) $$\frac{\partial D_{i}}{\partial r_{i}}=0$$

The second coupled wave equation is expressed as:

(7) $${ɛ}_{\text{ik}}^{S}\frac{\partial^{2}\phi }{\partial r_{i}\partial r_{k}}=e_{\text{ikl}}\frac{\partial^{2}u_{k}}{\partial r_{i}\partial r_{l}}$$

Equations (5) and (7) are two second order coupled wave equations in *i,j,k,l* = *x,y,z*. Solution of these equations yields four partial wave equations; displacement equations with polarizations in 3 directions and the voltage equation.

The solutions of the wave equations for a wave propagating along the *x*-direction with polarization in the *y*-direction are [27]:

(8) $$\begin{array}{l} u_{i}=\alpha_{i}\exp\;(\text{ikby})\exp\;(\text{ik}(x-\text{vt}))\\ \phi =\alpha_{i}\exp\;(\text{ikby})\exp\;(\text{ik}(x-\text{vt})) \end{array}$$

where (*b****)*** denotes the variation along the depth of the substrate and (*α*) is the amplitude of the wave, (*k*) is the wave number and (*v*) is the phase velocity. It is clear that the solutions of the displacement and voltage depend on the *x* and *y* dimensions only. No variation takes place along the *z* dimension. This is the plane wave solution, which can be used to reduce the size of the SAW sensor in the z-direction since the solution is independent of the thickness of the sensor. The SAW sensor can therefore be modeled using a reduced 3D model. This approach is adopted using the commercial FE Multiphysics package ANSYS® 12.0.

### 2.2. Frequency Response of SAW Devices

The frequency response of the SAW device is determined from the impulse response; both are Fourier transforms of each other:

(9) $$\begin{array}{l} h(t)=\int_{-\infty }^{\infty }H(f)e^{2\pi \text{ft}}\text{df}\\ H(f)=\int_{-\infty }^{\infty }h(t)e^{-2\pi \text{ft}}\text{dt} \end{array}$$

The delta function model approximates the frequency response of the SAW device as:

(10) $$\begin{array}{l} \left|H(f)\right|=N_{p}\left|\frac{\text{Sin}(X)}{X}\right|\\ \text{where};\\ X=N_{p}\pi \left(\frac{f-f_{o}}{f_{o}}\right) \end{array}$$

where *N_p_* is the number of electrode pairs of the IDT and *f_o_* is the centre frequency.

In this study a transient analysis is carried out to determine the impulse response of the SAW sensor from which the frequency response can be generated using an FFT code. To generate the impulse response an impulse signal is applied to the input set of electrodes, which has the following form:

(11) $$V_{\text{in}}=\{\begin{array}{lll} 1\times 10^{9} & \text{for} & 0<\text{t}\leq T_{s}\\ 0 & \text{for} & \text{t}>T_{s} \end{array}$$

where *T_s_* is the time step size, set to 1 ns.

## 3. Numerical Model of the SAW Sensor Using the Finite Element Method

The equations of piezoelectricity are fairly complex to allow a closed form solution and therefore, FE analysis is commonly used to provide an approximate solution to these equations using the variational and the virtual work principles. The virtual work per unit area created by surface tractions (*f*) due to a small virtual displacement (*u*) of the surface is {*δu*}*^t^* {*f*}. The electrical analog of the work due to the surface tractions (*f*) is the work created by the charge density (*q*) due to a virtual electric potential *φ*. The work due to charge density is expressed as −*q*{*δφ*}. The total virtual work done on the surface of the body is:

(12) $$\delta W={\left\{\delta u\right\}}^{t}\{F\}-\{\delta \phi \}\;\{q\}$$

The variational principle is expressed as:

(13) $$\delta \int_{t_{0}}^{t}\text{Ldt}+\int_{t_{0}}^{t}\delta W\;\text{dt}=0$$

The Lagrangian operator in this case consists of the difference between the kinetic energy and the electrical enthalpy L = E_Kin_ – H rather than the difference between the kinetic energy and the internal energy as in the case of pure elasticity [10]. The electrical enthalpy *H* is defined as the difference between the elastic energy (E_ST_) and the summation of the electro-mechanical (E_EM_) and dielectric energy (E_D_), H = E_ST_ – [E_EM_ + E_D_] [28].

The kinetic Energy is defined as:

(14) $$E_{\text{Kin}}=\frac{1}{2}∭\rho \dot{u}\dot{u}\text{dV}$$

and the elastic energy E_ST_ is defined as:

(15) $$E_{\text{ST}}=\frac{1}{2}∭{\left[S\right]}^{t}[c][S]\text{dV}$$

The Electro-mechanical coupling energy E_EM_ is defined as:

(16) $$E_{\text{EM}}=\frac{1}{2}∭{\left[S\right]}^{t}[e][E]\text{dV}$$

The dielectric energy E_D_ is defined as:

(17) $$E_{D}=\frac{1}{2}∭{\left[E\right]}^{t}[ɛ][E]\text{dV}$$

Expressing the work *W* in terms of body, surface and point loads and charges leads to:

(18) $$W=∭u^{t}f_{B}dV+\iint u^{t}f_{S}dA+\sum u^{t}f_{P}-\iint \varphi q_{s}\text{dA}-\sum \varphi q_{P}$$

where:

*f_b_*: mechanical body force vector (N/m^3^)

*f_s_*: mechanical surface force vector (N/m^2^)

*f_p_*: mechanical point forces (N)

*q_s_*: surface charges (C/m^2^)

*q_p_*: point charges (C)

In the FE formulation the body is discretized into finite elements, where the mechanical displacements *u*, electrical potential *φ*, electrical charge *q* and mechanical forces *f* are calculated at the nodes of these elements. The value at any position in the element is determined by means of linear combinations of polynomial interpolation functions *N* and the nodal values of these quantities as coefficients:

(19) $$u(x,y,z)=N_{u}(x,y,z)\cdot \hat{u}(x_{i},y_{i},z_{i})$$

(20) $$f_{B}(x,y,z)=N_{\text{FB}}(x,y,z)\cdot \hat{f}(x_{i},y_{i},z_{i})$$

(21) $$f_{S}(x,y,z)=N_{\text{FS}}(x,y,z)\cdot \hat{f}(x_{i},y_{i},z_{i})$$

Similarly, for the electric potential *φ* and electric charge *q*:

(22) $$\varphi (x,y,z)=N_{\varphi }(x,y,z)\cdot \hat{\varphi }(x_{i},y_{i},z_{i})$$

(23) $$q_{s}(x,y,z)=N_{\text{QS}}(x,y,z)\cdot \hat{q}(x_{i},y_{i},z_{i})$$

These expressions are then substituted in Equation (18) then into Equation (13). The Strain *S* and the electric field *E*, which are obtained by differentiating the displacement and the electric potential respectively can be expressed as:

(24) $$\begin{array}{l} S=\text{Bu}=B\left(N_{u}\hat{u}\right)=B_{u}\hat{u}\\ E=-\nabla \varphi =-\nabla \left(N_{\varphi }\hat{\varphi }\right)=B_{\varphi }\hat{\varphi } \end{array}$$

Substituting these expressions in Equation (14) up to Equation (17) then into Equation (13) yields the equilibrium equations

(25) $$M\ddot{u}+D_{\text{uu}}\dot{u}+K_{\text{uu}}u+K_{u\varphi }\varphi =F_{B}+F_{S}+F_{P}$$

(26) $$K_{u\varphi }^{t}u+K_{\varphi \varphi }\varphi =Q_{s}+Q_{P}$$

where the Matrices in Equation (25) and Equation (26) are defined as follows:

Mass Matrix [M]$\underset{V}{∭}\rho N_{u}^{t}N_{u}dV$Mechanical Stiffness Matrix [*K_uu_*]$\underset{V}{∭}B_{u}^{t}(c)B_{u}dV$Mechanical Body Force Matrix [*F_B_*]$\underset{V}{∭}N_{u}^{t}N_{\text{FB}}f_{B}dV$Mechanical Surface Force Matrix [*F_S_*]$\underset{A}{∭}N_{u}^{t}N_{\text{FS}}f_{s}dA$Mechanical Point Force Matrix [*F_P_*]$N_{u}^{t}f_{P}$Mechanical Damping Matrix [*D_uu_*]$\alpha^{*}∭\rho N_{u}^{t}N_{u}dV+\beta^{*}∭B_{u}^{t}c^{E}B_{u}dV$Piezoelectric Coupling Matrix [*K_uφ_*]$\underset{V}{∭}B_{u}^{t}(e)B_{\varphi }dV$Dielectric Stiffness Matrix [*K_φφ_*]$\underset{V}{∭}B_{\varphi }^{t}(ɛ)B_{\varphi }dV$Electrical Surface Charge Matrix [*Q_s_*]$-\iint_{S}N_{\varphi }^{t}N_{\text{QS}}q_{S}dA$Electrical Point Charge Matrix [*Q_P_*]$-N_{\varphi }^{t}q_{P}$*α and ***β*** are Damping coefficients

## 4. Reduced 3D Model (R3D) of Aluminum Nitride-Silicon (AlN/Si(111)) SAW Sensor

**Figure 3.**
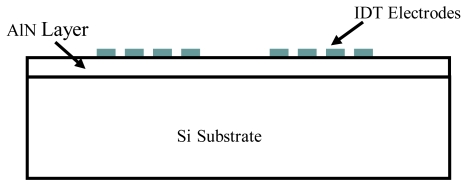
Schematic of the AlN/Si(111) SAW sensor.

The SAW device configuration adopted in this study consists of a silicon substrate with crystal axis orientation in the [111]. A thin aluminum nitride (AlN) film covers the silicon (Si) substrate with the electrodes placed on the free surface of the film. A schematic of the AlN-Si(111) SAW device is illustrated in Figure 3.

The elastic constants for silicon in the [100] direction are rotated accordingly to generate the properties in the [111] direction. The material properties of the Si(100) crystal are obtained from Madou [29]. Silicon has a cubic crystal structure and hence has three independent elastic constants; *c_11_* = 166, *c_12_* = 64, *c_44_* = 80 GPa and a density *ρ* = 2,320 Kg/m^3^. The material properties for aluminum nitride with density *ρ* = 3,260 Kg/m^3^ are listed in Table 1 (Tsubouchi [30]).

**Figure 4.**
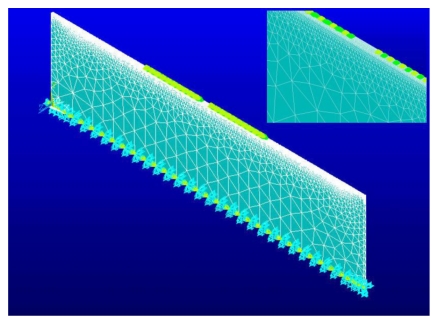
R3D FE model of AlN/Si(111). Inset illustrates the electrodes.

The FE model of the SAW sensor analyzed in this study is illustrated in Figure 4. The electrodes are modeled as a set of nodes coupled by a voltage degree of freedom. The electrodes in this model are assumed to have the same length in the z-direction as the thickness of the device. This corresponds to a case where a thin strip of the full 3D model is being analyzed.

### Boundary Conditions

**Figure 5.**
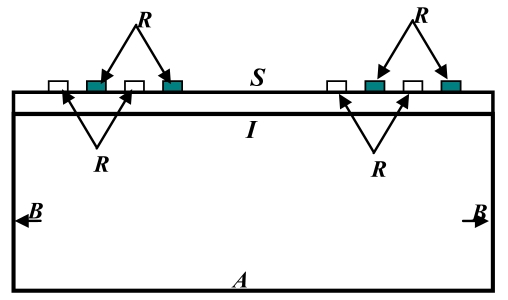
Boundary condition representation.

The boundary conditions adopted are listed below with respect to Figure 5.

- Clamped condition on the bottom surface *A*, to fix the sensor in place and reduce second order effects [7]:
  (27) $$\left(u_{x},u_{y},u_{z},\phi =0\right)$$
- Continuity of the displacement field components *U_i_ for i* = *x, y, z* at interface *I*.
- A Traction free boundary at the free surface *S* (z-plane):
  (28) $$T_{\text{iz}}=0;\;\text{for}\;i=x,y,z$$
- The following Dirichlet conditions for the electric potential:
  (28) $$\begin{array}{l} \phi {|}_{R1}=0\\ \phi {|}_{R2}=V(t)\\ \phi {|}_{R3}=O(t) \end{array}$$
  where *V(t)* is the input voltage signal and *O(t)* is the voltage response at the output electrodes.

Boundaries *B_1_* and *B_2_* are extended in the length direction as indicated by the arrows. This condition is necessary to avoid wave reflections from the boundaries that would cause interference and hence deteriorate the response.

Using the commercial FE Multiphysics simulation package ANSYS®12, the AlN film is meshed with a tetrahedral coupled Field element. There are four degrees of freedom per node, displacements *U_x_*, *U_y_*, *U_z_* and voltage *φ*. The silicon substrate is meshed with a tetrahedral structural field element, which has three degrees of freedom per node; *U_x_*, *U_y_* and *U_z_*. Based on mesh sensitivity analysis an element size of 2 μm is selected for elements used along the propagation path.

Various configurations of the FE model for the AlN/Si(111) SAW device are developed, each configuration adopts a different *h/λ* value. This is accomplished by maintaining a constant thickness of the AlN film (*h*) of 6 μm and varying the wavelength *λ* accordingly to achieve different *h/λ* values. The values of the wavelength *λ* for each case are listed in Table 3. As will be discussed later, increasing the thickness of the AlN layer increases the wave velocity. In addition, the wave velocity is related to the wavelength *λ* according to:

(30) v=fλ

where (*v*) is the SAW velocity in m/s and (*f*) is the centre frequency (Hz). A dimensionless parameter *h/λ* is introduced that relates to the wave velocity.

## 5. Results

Theoretical and experimental dispersion data for AlN/Si(111) are obtained from Caliendo *et al.* [26] and used to validate the current FE simulation results. The frequency response is obtained from the Fourier transform of the transient response and the values of the centre frequency are compared with the dispersion data from Caliendo *et al.* [26]. The parameters of the sensor for each case are similar except for the periodicity of the electrodes. The sensor parameters are listed in Table 2.

**Figure 6.**
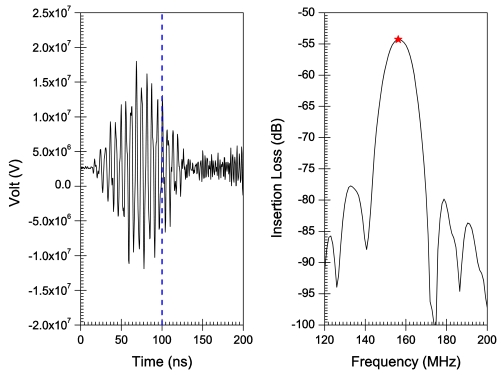
Time and frequency response for *h*/λ = 0.2.

**Figure 7.**
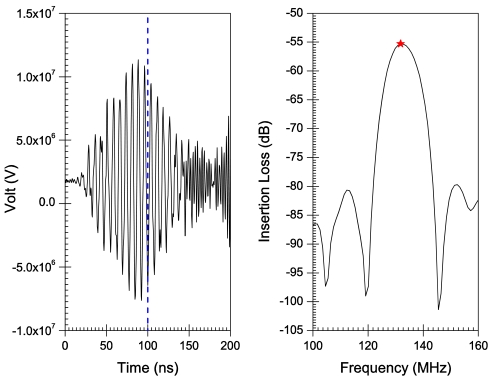
Time and frequency response for *h*/λ = 0.17.

**Figure 8.**
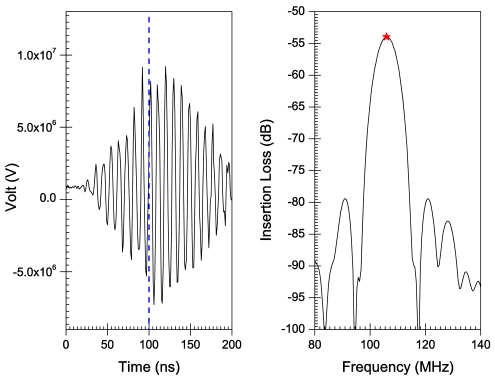
Time and frequency response for *h*/λ = 0.14.

**Figure 9.**
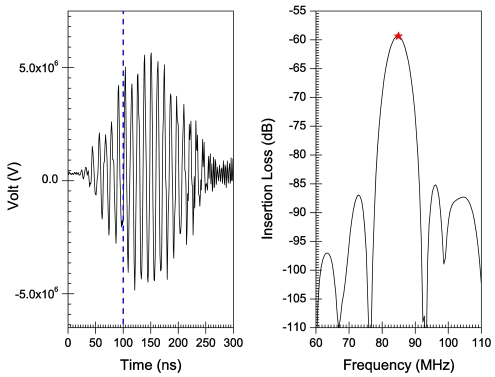
Time and frequency response for *h*/λ = 0.11.

**Figure 10.**
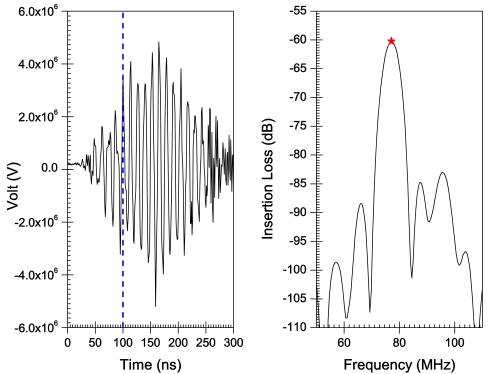
Time and frequency response for *h*/λ = 0.1.

**Figure 11.**
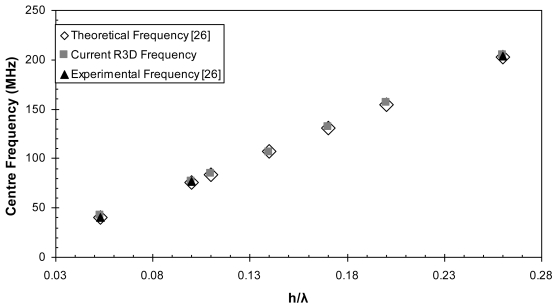
Comparison of the centre frequency values of the current R3D FE model with theoretical and experimental frequency data.

Figure 6–10 illustrate the time and frequency domain responses for the *h/λ* configurations from 0.1 to 0.2. The (★) in the frequency plots designate the centre frequency value. Figure 11 compares the centre frequency values of the current simulation with the experimental and theoretical frequency data reported in the literature for similar cases [26]. Table 3 provides a listing of the data plotted in Figure 11.

**Figure 12.**
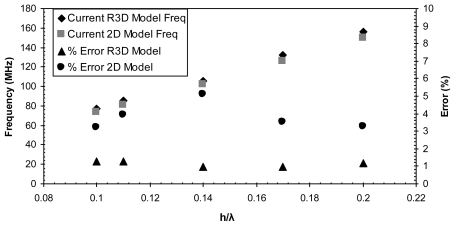
Comparing center frequency values of the current R3D FE model with the current 2D FE model.

**Figure 13.**
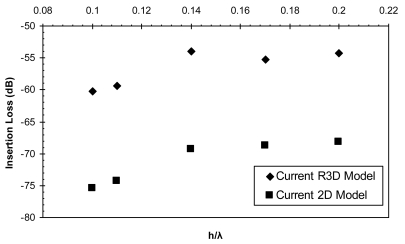
Comparison of insertion loss values between the R3D model and the 2D model.

Two dimensional models are developed for the AlN/Si(111) device and different configurations are adopted. The response of each configuration is compared with its equivalent configuration of the R3D model. The response from both models are compared in terms of their centre frequency values and their insertion loss values in Figure 12 and Figure 13 and the corresponding data are listed in Table 4 and Table 5, respectively.

## 6. Discussion

A transient analysis is carried out using FEA to obtain the time-domain response of AlN/Si(111) SAW sensors adopting different *h/λ* values. The frequency response is then obtained by Fourier transform of the transient response for each configuration. Response curves in time and frequency domains are illustrated in Figures 6–10 for *h/λ* values from 0.1 to 0.2. Hypothetical lines (---) are inserted in the transient response curves at 100 ns to illustrate the delay in wave speed that takes place due to decreasing *h/λ* values. For *h/λ* values of 0.2 and 0.17 the transient response reaches its peak prior to 100 ns, however as *h/λ* decreases the peak of the transient response shifts further away from 100 ns indicating a delay in the wave speed.

This behavior illustrates the dispersion property of the SAW wave, where the velocity of the wave changes accordingly with the thickness of the AlN film. As the SAW propagates along the surface of the layered AlN/Si(111) structure its velocity varies between that of AlN and silicon. The SAW velocity in AlN is higher than that in silicon; 5,607 and 4,550 m/s, respectively [31]. The increase in the *h/λ* parameter causes the wave to become more confined in the AlN layer, therefore its velocity increases until it eventually reaches that of AlN. The increase in wave velocity leads to an increase in the centre frequency of the SAW device as illustrated by Equation (30), which predicts a linear behavior. The centre frequency values for the different *h/λ* configurations are plotted in Figure 11 and a linear behavior is obtained as expected.

By adopting the plane wave solution Equation (8) the thickness of the sensor could be kept to a minimum while allowing polarizations in all three directions. Comparing the frequency response of the R3D model with the theoretical and experimental data shows very good agreement as illustrated in Figure 11. The reduced size of the model gives a higher flexibility in reducing the element size sufficiently along the propagation path and hence increasing the number of elements per wavelength to accurately capture the response.

The widely adopted approach in the literature is to develop 2D models in order to reduce the number of elements of the FE model significantly and reduce the required computational capacity. The main drawback in the 2D approach is that the displacement in the shear-horizontal direction is decoupled, which reduces the accuracy of the results. In order to demonstrate the impact of the R3D model, several 2D FE models of SAW devices with AlN/Si(111) layout were developed with similar *h/λ* values to the R3D models.

Figure 12 illustrates the frequency response of the R3D model in comparison with the 2D model for the different *h/λ* values. The error (%) with respect to the theoretical frequency values [26] is also plotted to illustrate the accuracy of both modeling approaches. The error (%) for the center frequency values of the R3D model are within 1%, however for the 2D model the error (%) varies between 3-5%. In addition, the insertion loss values of the R3D model and the 2D model are plotted in Figure 13. Results show a major discrepancy for all the *h/λ* values. The significant variations of the 2D model are due to the decoupling of the displacement component in the shear-horizontal direction.

## 7. Conclusions

In this study the FE theory for piezoelectric vibration is introduced and used to develop a new approach for simulating the performance of SAW sensors based on the plane wave solution. A reduced 3 dimensional model (R3D) is developed, where the thickness of the sensor was minimized to reduce the overall number of elements in the FE model and reduce the computational capacity. This approach allowed for wave polarizations in all three directions. The SAW device consisted of a thin AlN film that covered a Si(111) substrate. The response of the R3D model is compared with theoretical and experimental data points for different *h/λ* values and the results show very high agreement. In addition, 2D models were developed with similar *h/λ* values and the response is compared with the corresponding configuration of the R3D model. Centre frequency values were compared and the results of the R3D model showed a much lower error (%). Insertion loss values from both models were also compared and results of the 2D model shows major discrepancies. The results of the R3D model are very promising and demonstrate the significance of this approach in developing an accurate model without the need for extensive computational capacity.

## Figures and Tables

**Table 1.** Material properties of Aluminum Nitride.

**Elastic Matrix in Stiffness Form (× 10^11^ Pa)**		**Piezoelectric Matrix at Constant Strain(C/m^2^)**		**Permittivity Matrix at Constant Strain (×10^-11^ F/m)**	
*C_11_*	3.45	*e_15_*	–0.48	*ε_11_*	8
*C_12_*	1.25	*e_31_*	–0.58	*ε_33_*	9.5
*C_13_*	1.2	*e_33_*	1.55		
*C_33_*	3.95				
*C_44_*	1.18				
*C_66_*	$\frac{c_{11}-c_{12}}{2}=1.1$				

**Table 2.** Parameters of the AlN/Si(111) FE models adopted in this study.

**Parameter**	**Value**
Dimensions of the Si(111) Substrate	3,000 × 500 × 11 μm
Dimensions of the AlN Substrate	3,000 × 6 × 11 μm
Number of Electrode Pairs	10
Electrode width and Spacing	$\overset{\lambda }{\;}/\underset{4}{\;}$
Separation Distance	2λ
Thickness of AlN Film	6 μm

**Table 3.** Theoretical, simulation and experimental frequencyvalues for the different configurations.

***h/λ***	***h*(μm)**	**Lambda(λ) (μm)**	***V_Th_*(m/s)**	***F_Th_*(MHz)**	***F_Exp_* (MHz)**	***F_Sim_* (MHz)**
0.053	6	113.2	4567.62	40.35	40.03	42.48
0.1	6	60.00	4570	76.17	77.12	77.15
0.11	6	54.55	4575	83.88		84.96
0.14	6	42.86	4,586.67	107.02		105.96
0.17	6	35.29	4,608.3	130.57		131.84
0.2	6	30.00	4,633.33	154.44		156.25
0.26	6	23.00	4716.8	202.5	204.061	205.078

**Table 4.** Center frequency values for the R3D model and the 2D model for SAW devices with different *h*/λ values.

$\overset{h}{\;}/\underset{\lambda }{\;}$	Reduced 3D Model *F_sim_* (MHz)	2D Model *F_sim_* (MHz)
0.1	77.15	73.73
0.11	84.96	80.566
0.14	105.96	101.56
0.17	131.84	125.97
0.2	156.25	149.41

**Table 5.** Insertion loss values for the R3D model and the 2D model.

$\overset{h}{\;}/\underset{\lambda }{\;}$	Reduced 3D Model *IL*(dB)	2D Model *IL*(dB)
0.1	–60.245	-75.444
0.11	–59.363	-74.358
0.14	–53.98	-69.315
0.17	–55.30	-68.80
0.2	–54.30	-68.15

## References

[1] Liu Y., Cui T. (2007). Power Consumption Analysis of Surface Acoustic Wave Sensor Systems Using ANSYS and PSPICE. Microsyst. Technol..

[2] Anisimkin V.I., Penza M., Valentini A., Quaranta F., Vasanelli L. (1995). Detection of Combustible Gases by Means of a ZnO-on-Si Surface Acoustic Wave Delay Line. Sens. Actuat. B.

[3] Hoummady M., Bastien F. (1999). Acoustic Wave Viscometer. Rev. Sci. Instrum..

[4] Lee K., Wang W., Kim G., Yang S. (2006). Surface Acoustic Wave Based Pressure Sensor with Ground Sheilding Over Cavity on 41 YX LiNbO3. Jpn. J. Appl. Phys..

[5] Wu T.T., Chen Y.Y., Huang G.T., Chang P.Z. (2005). Evaluation of Elastic Properties of Submicrometer Thin Films Using Slanted Finger Interdigital Transducers. J. Appl. Phys..

[6] Gangadharan S., Varadan V.V., Jose K.A., Atashbar M.Z. Love Wave Based Ice Sensor.

[7] Campbell C. (1989). Surface Acoustic Wave Devices and Their Signal Processing Applications..

[8] Plessky V. (2000). Coupling of Modes Analysis of SAW Devices. Int. J. High Speed Electron. Syst..

[9] Tobolka G. (1979). Mixed Matrix Representation of SAW Transducers. IEEE Trans. Sonics Ultrason..

[10] Allik H., Hughes T. (1970). Finite Element Method for Piezoelectric Vibration. Int. J. Numer. Methods Eng..

[11] Lerch R. (1990). Simulation of Piezoelectric Devices by Two- and Three-Dimensional Finite Elements. IEEE Trans. Ultrason., Ferroelectr. Freq. Control.

[12] Atashbar M.Z., Bazuin B.J., Simpeh B.M., Krishnamurthy S. 3-D Finite Element Simulation Model of SAW Palladium Thin Film Hydrogen Sensor.

[13] Ippolito S.J., Kalantar-Zadeh K., Wlodarski W., Matthews G.I. The Study of ZnO/XY LiNbO_3_ Layered SAW Devices for Sensing Applications.

[14] Botkin N., Schlensog M., Tewes M., Turova V. A Mathematical Model of a Biosensor.

[15] Xu G. (2000). Direct finite-element analysis of the frequency response of a Y-Z lithium niobate SAW filter. Smart Mater. Struct..

[16] Xu G., Jiang Q. (2001). A finite element analysis of second order effects on the frequency response of a SAW device. J. Intell. Mater. Syst. Struct..

[17] Abdollahi A., Jiang Z., Arabshahi S.A. (2007). Evaluation on Mass Sensitivity of SAW Sensors for Different Piezoelectric Materials Using Finite-Element Analysis. IEEE Trans. Ultrason., Ferroelectr. Freq. Control..

[18] Ippolito S.J., Kalantar-Zadeh K., Powell D.A, Wlodarski W. (2003). A 3-Dimensional Approach for Simulating Acoustic Wave Propagation in Layered SAW Devices.

[19] Atashbar M.Z., Bazuin B.J., Simpeh B.M., Krishnamurthy S. (2005). 3D FE Simulation of H2 SAW Gas Sensor. Sens. Actuat. B.

[20] Wong K.Y., Tam W.Y. (2005). Analysis of the Frequency Response of SAW Filters using Finite-Difference Time-domain Method. IEEE Trans. Microwave Theory Tech..

[21] Wu S., Wu L., Chang J.H., Chang F.C. (2001). SAW Modes on ST-X Quartz with an AlN Layer. Mat. Lett..

[22] Jin Y., Joshi S. (1996). Propagation of a Quasi-Shear Horizontal Acoustic Wave in Z-X Lithium Niobate Plates. IEEE Trans. Ultrason. Ferroelectr. Freq. Control.

[23] Powell D.A., Zadeh K.K., Wlodarski W. (2004). Numerical Calculation of SAW Sensitivity: Application to ZnO/LiTaO3 Transducers. Sens. Actuat. A.

[24] Hietala V.M., Casalnuovo S.A., Heller E.J., Wendt J.R., Frye-Mason G.C., Baca A.G. (2000). Monolithic GaAs Surface Acoustic Wave Chemical Microsensor Array.

[25] Hickernell F.S., Zinc Oxide (1985). Films for Acoustoelectric Device Applications. IEEE Trans. Sonics Ultrason..

[26] Caliendo C., Imperatori P., Cianci E. (2003). Structural Morphological and Acoustic Properties of AlN Thick Films Sputtered on Si(001) and Si(111) Substrates at Low Temperature. Thin Solid Films.

[27] Mason W.P. (1972). Physical Acoustics Principles and Methods.

[28] Tiersten H.F. (1967). Hamilton's Principle for Linear Piezoelectric Media. Proc. IEEE..

[29] Madou M.J. (2002). Fundamentals of Microfabrication..

[30] Tsubouchi K., Mikoshiba N. (1985). Zero Temperature Coefficient SAW Devices on AlN Epitaxial Films. IEEE Tran. Sonics Ultrason..

[31] Caliendo C., Verona E., Cimmino A. (2001). Microwave frequency acoustic resonators implemented on monolithic Si/AlN substrates. Proc. SPIE..

